# Disjoint motif discovery in biological network using pattern join method

**DOI:** 10.1049/iet-syb.2019.0008

**Published:** 2019-07-04

**Authors:** Sabyasachi Patra, Anjali Mohapatra

**Affiliations:** ^1^ Department of Computer Science IIIT Bhubaneswar Odisha India

**Keywords:** graph theory, bioinformatics, network theory (graphs), complex networks, pattern classification, microorganisms, proteins, computational complexity, diseases, molecular biophysics, genetics, Saccharomyces cerevisiae, Escherichia coli, pattern join method, disjoint motif discovery, protein interaction network, transcription regulatory network, target network, network motif, biological network

## Abstract

The biological network plays a key role in protein function annotation, protein superfamily classification, disease diagnosis, etc. These networks exhibit global properties like small‐world property, power‐law degree distribution, hierarchical modularity, robustness, etc. Along with these, the biological network also possesses some local properties like clustering and network motif. Network motifs are recurrent and statistically over‐represented subgraphs in a target network. Operation of a biological network is controlled by these motifs, and they are responsible for many biological applications. Discovery of network motifs is a computationally hard problem and involves a subgraph isomorphism check which is NP‐complete. In recent years, researchers have developed various tools and algorithms to detect network motifs efficiently. However, it is still a challenging task to discover the network motif within a practical time bound for the large motif. In this study, an efficient pattern‐join based algorithm is proposed to discover network motif in biological networks. The performance of the proposed algorithm is evaluated on the transcription regulatory network of *Escherichia coli* and the protein interaction network of *Saccharomyces cerevisiae*. The running time of the proposed algorithm outperforms most of the existing algorithms to discover large motifs.

## 1 Introduction

Network motifs are basic building blocks of various biological networks such as metabolic network, gene regulatory network, and protein interaction network [1]. These are not only studied in a biological network, but also key features in many other networks such as social network, ecological network (food web), World Wide Web (the Internet), etc. Network motifs are over‐represented patterns in a target network like a sequence motif in a protein sequence. But network motif discovery requires computationally expensive isomorphic testing and repeated frequency computation for the statistical significance measure. Network motifs act as a key feature in a wide range of applications of biological networks. Most of the biological networks possess two critical motifs: feed‐forward‐loop and Bifan [2]. However, motifs like autoregulation, feedback loops, and dense overlapping regulons, etc. [3] are functionally important. Przulj *et al.* [4] distinguish different protein–protein interaction networks by using network motifs as a feature. These are also used for network model selection. Based on motif significance profiles, Milo *et al.* [5] classified networks of the various domains into superfamilies. Albert and Albert [6] used these features successfully to predict protein–protein interactions. Gupta *et al.* [7] used network motifs for cancer disease diagnosis. These are also used for network superfamily classification [5] and artificial network model for a real‐world network, prediction of breast cancer survival outcome, analysis of functional network in diabetes patients, etc. A three‐node network motif found in the human waving network helps recognise breast cancer patients from regular patients [8].

Network motif discovery algorithms broadly classified into two categories: (i) network‐centric and (ii) motif‐centric [9]. Depending on frequency computation again, they can be classified as exact search and sampling. Some of the network‐centric algorithms are enumerate subgraphs (ESU) [10], MFinder [11], MAVisto [12], NeMoFinder [13], Kavosh [14] and FANMOD [15]. Out of these algorithms, MFinder and FANMOD use a sampling approach for counting motif frequency, whereas other algorithms use the exact census. Two popular motif centric algorithms are Grochow and Kellis [16] and MODA [17]. Both of these algorithms follow the exact census approach. A brief introduction to some of the existing algorithms is given in the next paragraph.

The first significant contribution in network motif discovery by Milo *et al.* [1], published in 2002. To measure the statistical significance, the frequency of a motif in a real network is compared with a set of random networks having the same degree distribution as the real network. A backtracking algorithm name as MFinder is used for discovering network motifs. The exponential space complexity of this algorithm made this method incapable of dealing with large motifs. Kashtan *et al.* [18] improved the execution time of motif detection algorithm by sampling approach, but the results obtained are biased. Wernicke [10] proposes a specialised algorithm ESU that could avoid redundancy in computation through proper enumeration. This method uses a third‐party algorithm NAUTY [19] for checking isomorphism. A lot of redundant subgraph isomorphism check is involved in this method as it is not able to handle automorphism. The flexible pattern finder algorithm [20] proposed a pattern growth approach for computing pattern frequency. However, the number of patterns grows rapidly concerning increase pattern size. Therefore, searching all patterns systematically is a time‐consuming task, even for a medium‐size pattern. Grochow and Kellis [16] proposed a motif centric algorithm, where frequency counting is done on a specific isomorphic class. This algorithm avoids unnecessary and redundant searches by mapping the query graph only on one representative of its equivalence class. The symmetry conditions are removed by adding constraints on the labelling of the vertices. These conditions reduce the number of isomorphic checks significantly. However, subgraph isomorphism is still a significant concern in this method. Kashani *et al.* [14] proposed a new network‐centric algorithm named as Kavosh. This algorithm generates all combinations with the desired number of nodes through an implicit tree rooted at the chosen vertex. Omidi *et al.* [17] proposed MODA, which is based on a pattern growth methodology. This is a subgraph‐centric algorithm. The core idea of this algorithm is first to find the frequency of acyclic subgraphs, save the respective embeddings in memory and then use those embeddings to quickly find out the frequencies of cyclic subgraphs. MODA introduces the concept of expansion tree, which is static and built at the beginning of the algorithm. A novel algorithm named as CoMoFinder proposed by Liang *et al.* [21]. Composite network motifs present in co‐regulatory networks are identified accurately and efficiently by this method. Parallel subgraph enumeration strategy is applied to this method. Elhesha and Kahveci [22] proposed a motif centric algorithm for finding motifs in a target network. The core idea of this method is to build a set of basic building patterns and find instances of these patterns. Then the size of the motifs increases by joining the known motifs with the instances of basic building patterns. Lin *et al.* [23] used Graphical Processing Units (GPUs) to study network motif. GPUs are employed to parallelise subgraph matching tasks in random graphs, which significantly reduce the overall computation time. Chen and Chen [24] published an efficient sampling algorithm for network motif detection.

The existing methods face significant challenges when the motif size increases [25, 26]. The performance of most of the existing algorithms that follow the exact census significantly decreases with increase motif size. The performance of algorithms which follow sampling approaches is biased and hence unreliable. Further, some methods are applicable only for finding overlapping motif instances. Network motif discovery in a large and complex biological network is time consuming, as the number of alternative motif topologies increases exponentially and it involves a subgraph isomorphism check. Furthermore, the number of alternative topologies increases exponentially with the increase of subgraph size. For this reason, existing methods only focus on motifs of small size. This limitation prevents further investigation in this field. In this paper, we adopt a pattern join method to identify large network motifs in a biological network efficiently. The central idea of this algorithm is to use some basic building patterns and find their embeddings. This is followed by an iterative joining of parent patterns with these basic building patterns. As a result, child patterns of higher order are obtained. Non‐overlapping motif instances are obtained by using the maximum independent set (MIS) finding [22] algorithm. The proposed algorithm significantly reduces the computationally expensive isomorphic test and avoids unnecessary growth of pattern which does not have any statistical significance.

The remaining of the paper is organised as follows: Section 2 presents an overview of the motif discovery process. Section 3 presents the proposed network motif discovery algorithm. Implementation, results, and discussion are presented in Section 4. Finally, Section 5 presents a brief conclusion with the future scope of this paper.

## 2 Network motif discovery process

**Fig. 1:**
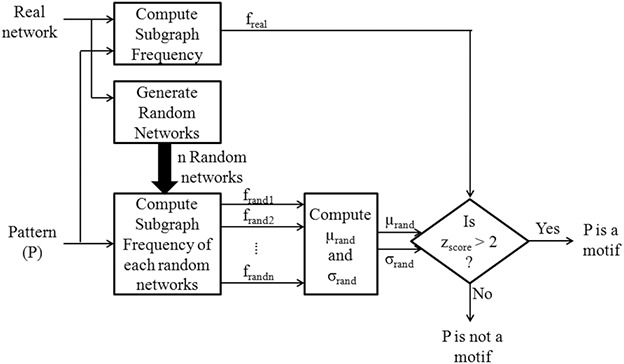
Block diagram of network motif discovery process

**Fig. 2:**
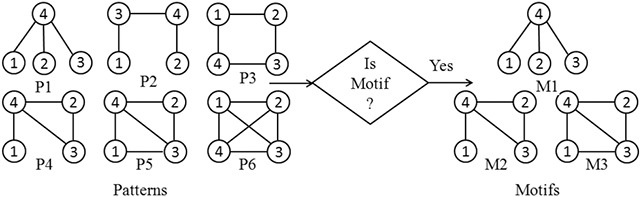
Demonstration of the presence of size‐4 significant motifs in a hypothetical network

Network motif discovery is the process of finding statistically significant patterns within a target network. The target network and all the potential motifs are represented as graphs. The subgraph in a graph with a frequency higher than the predefined threshold is considered to be a potential motif. The major steps in the network motif discovery process consist of (i) pattern frequency computation, (ii) random graph generation, and (iii) statistical testing. The block diagram of the motif discovery process is shown in Fig. 1. In Fig. 2, hypothetical data demonstrates that out of six non‐isomorphic subgraphs of size‐4, three patterns are determined as network motifs.

**Fig. 3:**
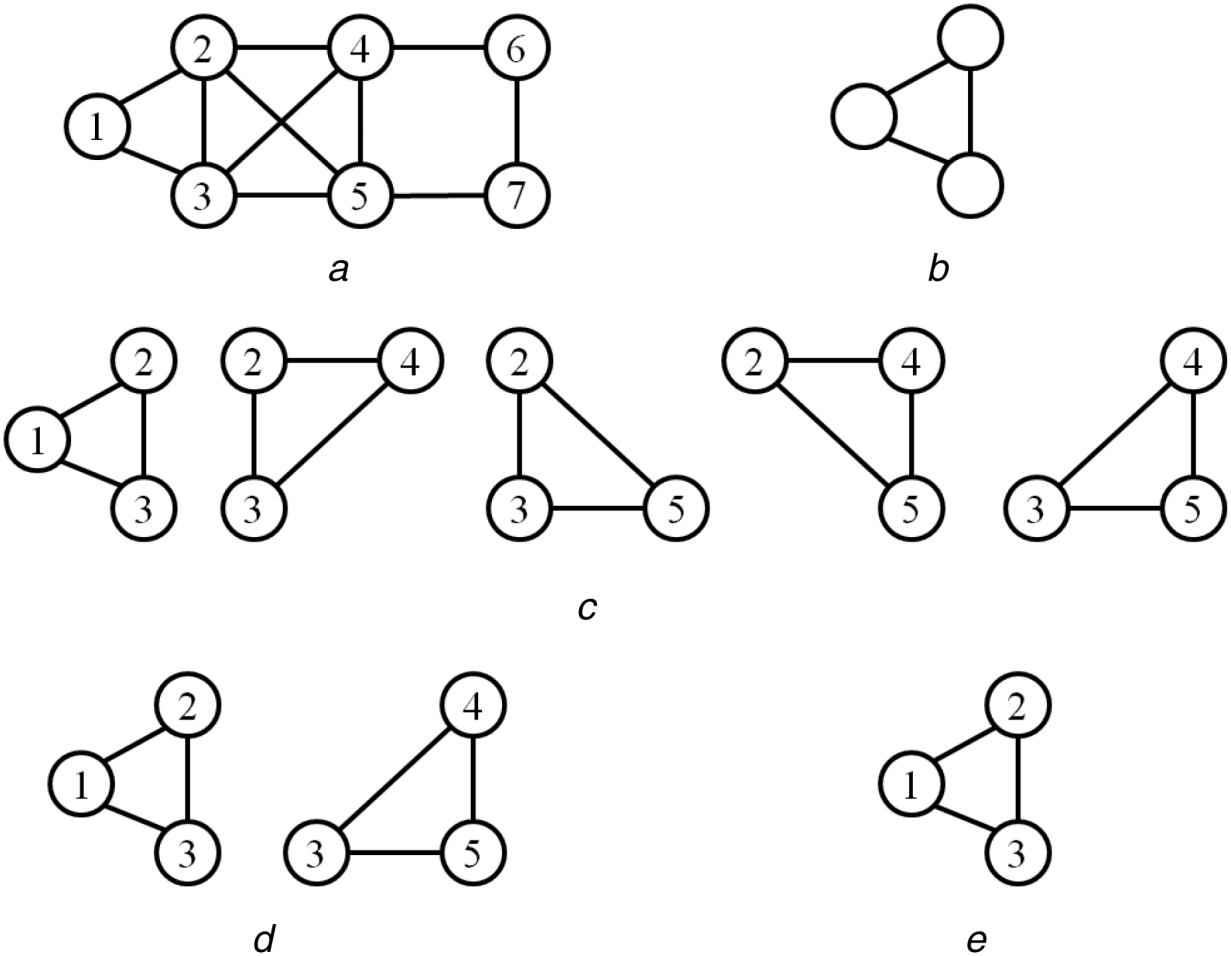
Illustration of different frequency concepts ***(a)*** Target network, ***(b)*** Size‐3 candidate motif, ***(c)***–***(e)*** Embeddings with respect to frequency measures *F*1, *F*2 and *F*3, respectively

The frequency of patterns in a target network is measured by using three different frequency measures *F*1, *F*2, and *F*3. These frequencies are defined concerning the overlapping of graph elements in subgraph instances. *F*1 measure, both vertices and edges can be shared among different instances of the subgraph. *F*2 measure computes edge‐disjoint instances of the subgraph where only vertices can be shared. *F*3 measure is completely restrictive, in which no sharing of vertices or edges are allowed. Frequency measure *F*2 is used in the proposed algorithm as it counts edge‐disjoint subgraphs, which satisfy downward closure property [27]. The downward closure property ensures that the frequency of child patterns (i.e. patterns obtained from parent after join operation) is monotonically decreasing with increasing size of the pattern. Based on this property, the search space of patterns can be reduced by pruning of infrequent patterns in the iterative joining process. Hence it reduces the search space for finding frequent patterns and therefore ensures fast computation. In Fig. 3, a hypothetical network and a size‐3 candidate motif with all its embeddings for different frequency measures are shown.

**Fig. 4:**
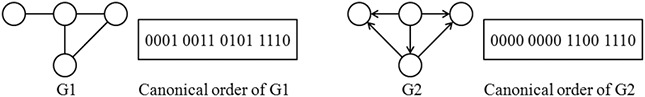
Graphs with canonical order

Graph isomorphism check plays a significant role in motif frequency computation. The fastest way to check graph isomorphism is through canonical ordering or canonical labelling. The vertices of a graph are assigned with a unique label in canonical order that makes it invariant under isomorphism. If two or more graphs have the same canonical labelling, then they are guaranteed to be isomorphic with each other. Canonical ordering is obtained by using McKay's canonical graph labelling algorithm (Nauty tool) [19]. An undirected graph and a directed graph with their canonical order are shown in Fig. 4.

**Fig. 5:**
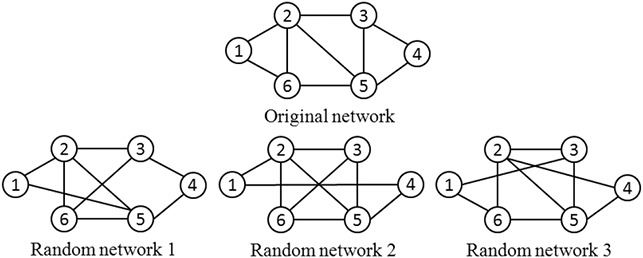
Random networks preserving degree distribution of the original network

Another essential step in a network motif discovery process is generating random networks, which is used to measure the statistical significance of a motif in a target network. The generated random networks must possess the same properties as the target network, such as the number of edges, the number of nodes, and the degree distribution of nodes, etc. In creating the random network, there exist two common algorithms: (i) matching algorithm and (ii) switching algorithm. A hypothetical network and some randomly generated networks, which preserve the required properties are shown in Fig. 5.

The last key step in the motif discovery process is a statistical significance measure of a potential motif. There are three important measures such as *P*‐value, *z*‐score, and significance profile (SP) used for this purpose. The *z*‐score of a motif *M* is defined as $z(M)=(f_{\text{real}}-\mu_{\text{rand}})/\sigma_{\text{rand}}$, where $f_{\text{real}}$ is the frequency of motif in the target network, $\mu_{\text{rand}}$ and $\sigma_{\text{rand}}$ are the mean frequency and standard deviation of frequencies of a set of random networks. The *P*‐value of a motif is defined as n/N, where *n* is the number of times $f_{\text{rand}}\geq f_{\text{real}}$ and *N* is the total number of random networks. A vector representing the *z*‐scores of a set of motifs is called a significance profile (SP). Motifs found in a biological network are not necessarily functionally important. But they are always statistically significant.

## 3 Network motif discovery using the pattern‐join method

**Fig. 6:**
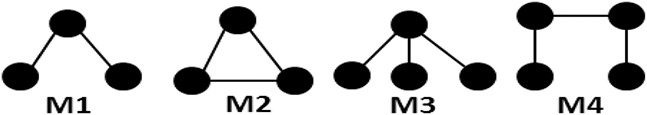
Basic building patterns for undirected graph

**Fig. 7:**
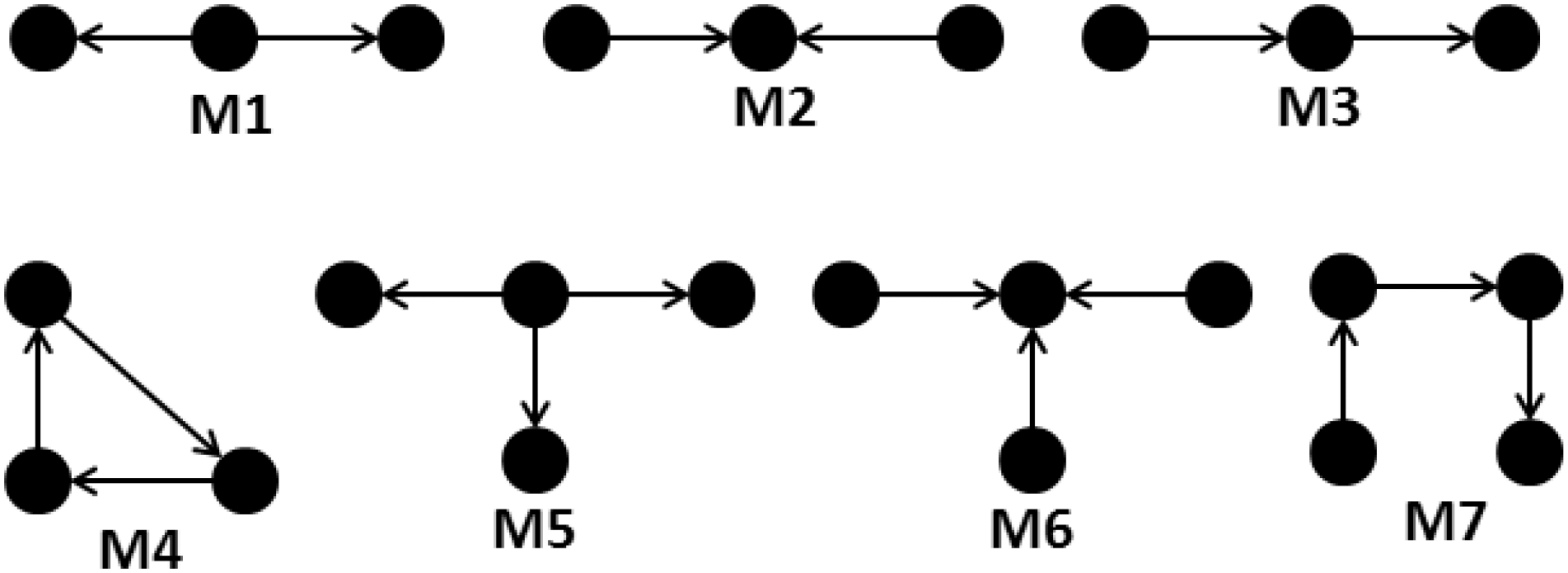
Basic building patterns for directed graph

In this paper, we adopt a pattern join method to identify large network motifs in biological networks efficiently. The central idea of this algorithm is to use some basic building patterns and find their embeddings. This is followed by an iterative joining of parent patterns with these basic building patterns. As a result, child patterns of higher order are obtained. The proposed algorithm significantly reduces the computationally expensive isomorphic test and avoids unnecessary growth of pattern, which does not have any statistical significance. A proposed motif discovery algorithm is a motif centric algorithm. The basic patterns can generate all possible patterns through iterative joining and hence called basic building patterns. The basic building patterns of undirected and directed graphs are shown in Figs. 6 and 7, respectively. There are four basic building patterns selected for an undirected graph, and seven basic building patterns are selected for a directed graph. The proposed algorithm initialises the current set of patterns with these basic building patterns. Iteratively, each pattern present in the current set is joined with basic building patterns to construct a new set of patterns. At the end of an iteration, the new set of patterns becomes the current set for the next iteration.

**Fig. 8:**
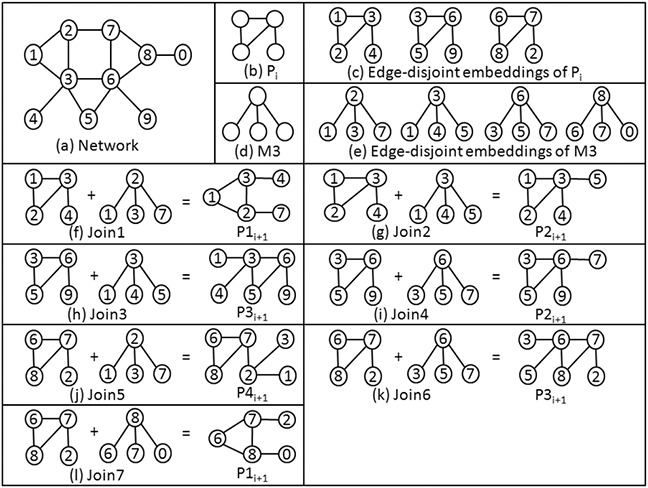
Pattern‐join operation in undirected graph ***(a)*** Hypothetical network, ***(b)*** Pattern $P_{i}$, ***(c)*** Edge‐disjoint embeddings of $P_{i}$, ***(d)*** Basic building pattern M3, ***(e)*** Edge‐disjoint embeddings of basic building pattern M3, ***(f)***–***(l)*** Joining operation of embeddings of pattern $P_{i}$ with embeddings of basic building pattern M3 results four new patterns $P1_{i+1}$, $P2_{i+1}$, $P3_{i+1}$, and $P4_{i+1}$. Join‐1 and join‐7 produce the same patterns because the resultant graph are isomorphic with each other. Similarly, join‐2 and join‐4 result the same patterns and join‐3 and join‐6 result the same patterns. Subscript *i* represents the pattern in the *i*th iteration and subscript i+1 represents the resultant pattern in the i+1 iteration

**Fig. 9:**
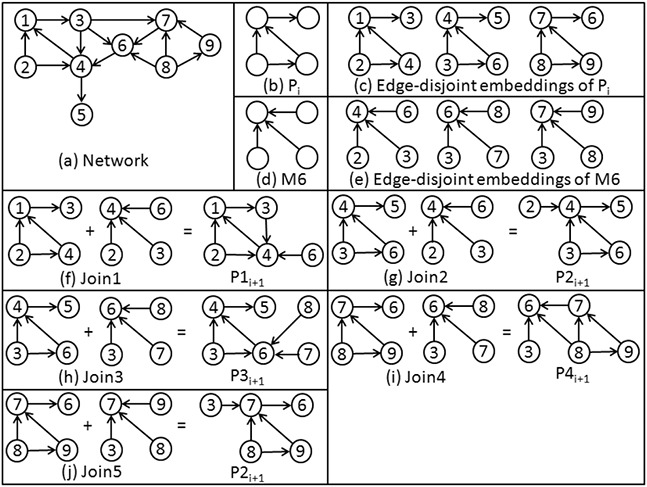
Pattern‐join operation in directed graph ***(a)*** Hypothetical network, ***(b)*** Pattern $P_{i}$, ***(c)*** Edge‐disjoint embeddings of $P_{i}$, ***(d)*** Basic building pattern M6, ***(e)*** Edge‐disjoint embeddings of basic building pattern M6, ***(f)***–***(j)*** Joining operation of embeddings of pattern $P_{i}$ with embeddings of basic building pattern M6 results four new patterns $P1_{i+1}$, $P2_{i+1}$, $P3_{i+1}$, and $P4_{i+1}$. Join‐2 and join‐5 produce the same patterns because the resultant graphs are isomorphic with each other. Subscript *i* represents the pattern in the *i*th iteration and subscript i+1 represents the resultant pattern in the i+1 iteration

During the joining process, two subgraphs can be joined if they share at least one edge. To avoid unnecessary checking on join operation, self‐joining is not allowed in the proposed algorithm. As a result, computational cost decreases. The proposed method finds the disjoint motif instances, and self‐joining will never happen on disjoint motif instances. The joining of two subgraphs either yields an existing subgraph in the new set or a new subgraph. Existing subgraph generated is treated as a duplicate subgraph and discarded by the proposed algorithm. The pattern of the newly created subgraph is either isomorphic to one of the existing patterns or a new one. In the former case, we consider the generated subgraph as an embedding of its corresponding pattern, and the algorithm increments the pattern frequency. In the case of the new pattern, it is added to the current set, and its frequency is initialised to 1. Subgraph isomorphism is checked by comparing the canonical order of subgraph with the canonical order of all the patterns present in the current set. Nauty toll [19] is used for this purpose. Figs. 8 and 9 demonstrate the pattern‐join operation in the undirected graph and directed graph, respectively.

The critical observations in this pattern‐join method are

- The two sets of basic building patterns, as shown in Figs. 6 and 7 are unique. There exist no other equivalent set of basic patterns. Therefore, these two sets represent minimal or irreducible sets of patterns.
- If anyone of the patterns is removed from the set of basic building patterns, then that cannot be generated without self‐joining.
- Any pattern with *k* + 1 edges can be obtained from a parent pattern with *k* edges by joining with one of the basic patterns.

The above observations are further justified below.

**Fig. 10:**
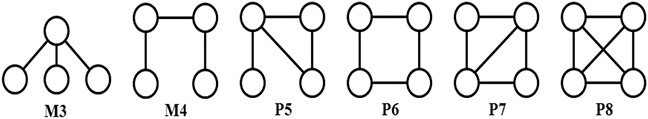
All possible order‐4 patterns for undirected graph

**Fig. 11:**
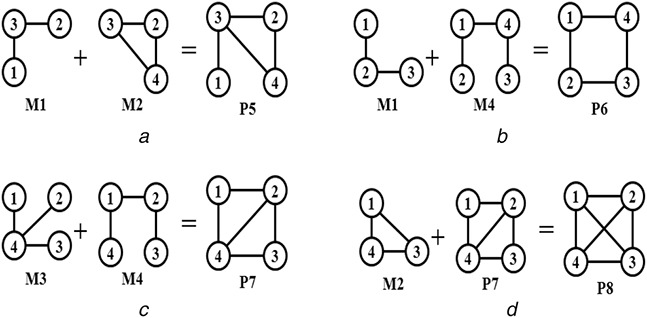
Generation of order‐4 patterns using pattern join operation for undirected graph

Let us consider the basic building patterns of the undirected graph first. The minimum order (number of vertices) of the basic pattern is chosen as 3. Because the order‐2 graph represents an edge and the method will no longer be a pattern‐join method. It will be a simple edge addition process. There are two possible patterns of order‐3, represented by M1 and M2, as shown in Fig. 6. Both M1 and M2 must be considered in the basic building patterns as one cannot be generated from others without self‐joining. Now consider all possible connected patterns of order‐4 as shown in Fig. 10. M3 cannot be generated from M1 and M2 without self‐joining, and M4 cannot be generated from M1, M2, and M3 without self‐joining. Hence M3 and M4 must be included in the set of basic building patterns. Now consider an instance of P5 that can be generated by joining instances of M1 and M2 as shown in Fig. 11*a*. An instance of P5 can also be generated by joining instances of M1 and M3 or M1 and M4 or M2 and M3 or M2 and M4 or M3 and M4. Similarly, instances of P6, P7, and P8 can be generated by joining instances of basic patterns among themselves or by joining an instance of basic pattern with an instance of the already generated pattern. Generation of an instance from each of the above patterns is shown in Fig. 11. The pattern of higher order can be generated by the pattern‐join operation as stated in the third observation, and that is explained below.

**Fig. 12:**
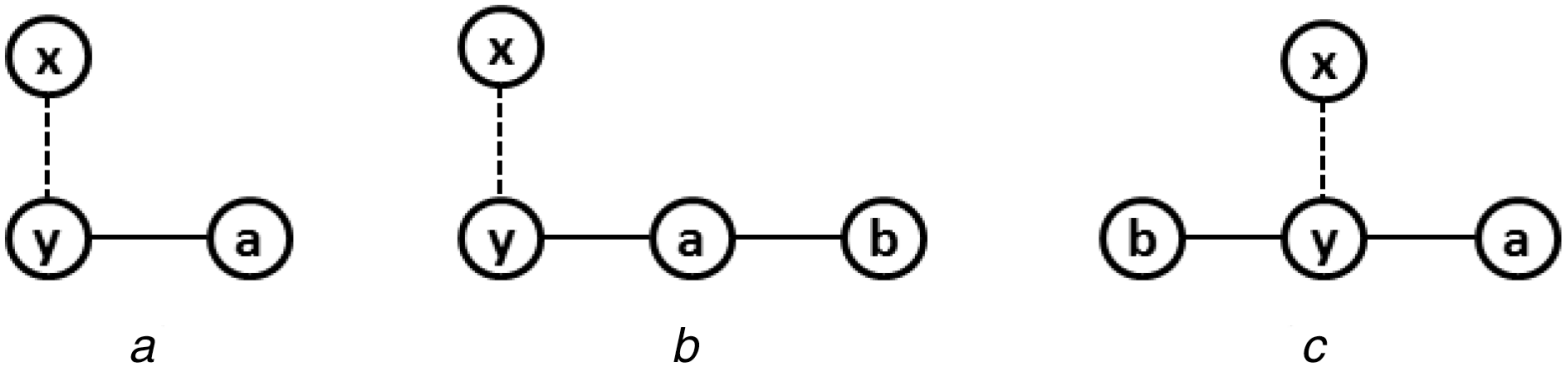
Generation of a pattern with k + 1 edges from a pattern with k edges. (x, y) is the additional edge in the child pattern ***(a)*** Assuming an existing edge (*y*, *a*) in the parent pattern, the child pattern is generated as a result of joining the parent pattern with the subgraph {(*x*, *y*), (*y*, *a*)} which belongs to M1 (see Fig. 6). Failure to accomplish the join in (a), either ***(b)*** child pattern is obtained by joining the parent pattern with the subgraph {(*x*, *y*), (*y*, *a*), (*a*, *b*)} which belongs to M4 (see Fig. 6) or ***(c)*** it is generated by joining the parent pattern with the subgraph {(*x*, *y*), (*a*, *y*), (*b*, *y*)} which belongs to M3 (see Fig. 6)

Let us consider an undirected graph *G* and pattern P1 of size *k* edges in *G*. Also, consider pattern P2 with *k* + 1 edges such that P2 contains P1 and an additional edge (*x*, *y*). It is required to show that P2 can be obtained from P1 by joining it with one of the four basic building patterns. Since both P1 and P2 are connected graphs, let us assume that *y* has an edge (*y*, *a*) present in pattern P1. Fig. 12 illustrates the two edges (*x*, *y*) and (*y*, *a*). First, basic building pattern M1 (Fig. 6) is considered for the join operation. In this case, a copy of M1, {(*x*, *y*), (*y*, *a*)} and pattern P1 joined together to form pattern P2. However, this join occurs only if the subgraph {(*x*, *y*), (*y*, *a*)} is included in the *F*2 counts of M1. If the above condition fails, then depending on the degree of the nodes *y* and *a* in pattern P1, there may exist an edge (*y*, *b*) or (*a*, *b*) as shown in Fig. 12. If (*a*, *b*) exist then join a copy of the motif M4 (Fig. 6), {(*x*, *y*), (*y*, *a*), (*a*, *b*)} with P1 to obtain P2. Otherwise, if (*y*, *b*) exist then join a copy of the motif M3 (Fig. 6), {(*y*, *x*), (*y*, *a*), (*y*, *b*)} with P1 to obtain P2.

**Fig. 13:**
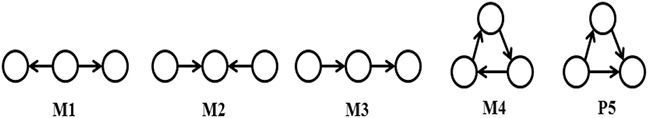
All possible order‐3 digraph patterns

**Fig. 14:**
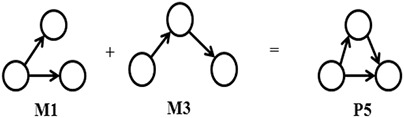
Generation of order‐3 digraph pattern P5 from M1 and M3

Now consider the basic building patterns of the directed graph. Similar to the undirected graph, the order of basic building patterns for a directed graph is also started with 3. Because the order‐2 graph represents an edge and it leads to edge addition process in place of the pattern‐join operation. Let us consider all possible connected patterns of order‐3, as shown in Fig. 13. The patterns M1, M2, M3, and M4, must be considered in the basic building patterns as one cannot be generated from others without self‐joining. However, P5 can be generated by joining an instance of M1 with M3, as shown in Fig. 14. Thus P5 is not included in the basic building patterns.

**Fig. 15:**
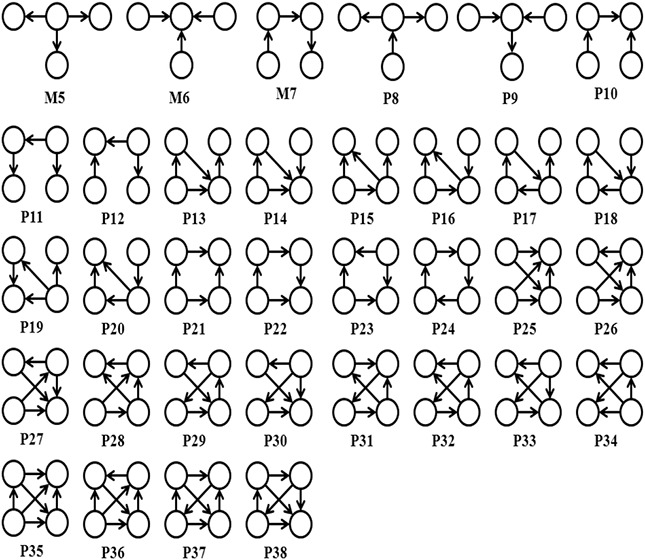
All possible order‐4 digraph patterns

Now consider all possible digraph patterns of order‐4 as shown in Fig. 15. M5 cannot be generated from M1, M2, M3, and M4 without self‐joining and M6 cannot be generated from M1, M2, M3, M4, and M5 without self‐joining and M7 cannot be generated from M1, M2, M3, M4, M5, and M6 without self‐joining. Hence M5, M6, and M7 must be included in the set of basic building patterns. Now consider an instance of P8 that can be generated by joining instances of M1 and M3. An instance of P9 and P10 can be generated by joining instances of M2 and M3. An instance of P11 can be generated by joining instances of M1 and M3. An instance of P12 can be generated by joining instances of M1 and M2. Similarly, instances of P13–P38 can be generated by joining instances of basic patterns among themselves or by joining an instance of basic pattern with an instance of the already generated pattern. The pattern of higher order can be generated by the pattern‐join operation as stated in the third observation and that is explained below.

**Fig. 16:**
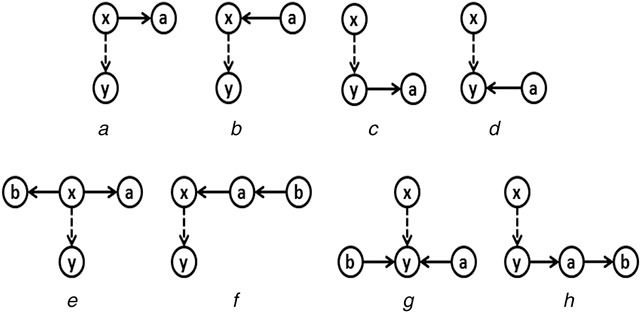
Generation of a pattern with k + 1 edges from a pattern with k edges. (x, y) is the additional edge in the child pattern ***(a)*** Assuming an existing edge (*x*, *a*) in the parent pattern, the child pattern is generated as a result of joining the parent pattern with the subgraph {(*x*, *y*), (*x*, *a*)} which belongs to M1 (see Fig. 7), ***(b)*** Assuming an existing edge (*a*, *x*) in the parent pattern, the child pattern is generated as a result of joining the parent pattern with the subgraph {(*a*, *x*), (*x*, *y*)} which belongs to M3 (see Fig. 7), ***(c)*** Assuming an existing edge (*y*, *a*) in the parent pattern, the child pattern is generated as a result of joining the parent pattern with the subgraph {(*x*, *y*), (*y*, *a*)} which belongs to M3 (see Fig. 7), ***(d)*** Assuming an existing edge (*a*, *y*) in the parent pattern, the child pattern is generated as a result of joining the parent pattern with the subgraph {(*x*, *y*), (*a*, *y*)} which belongs to M2 (see Fig. 7). Failure to accomplish the above joins, either ***(e)*** a child pattern is obtained by joining the parent pattern with the subgraph {(*x*, *y*), (*x*, *a*), (*x*, *b*)} which belongs to M5 (see Fig. 7) or ***(f)*** it is generated by joining the parent pattern with the subgraph {(*b*, *a*), (*a*, *x*), (*x*, *y*)} which belongs to M7 (see Fig. 7) or ***(g)*** it is generated by joining the parent pattern with the subgraph {(*a*, *y*), (*b*, *y*), (*x*, *y*)} which belongs to M6 (see Fig. 7) or ***(h)*** it is generated by joining the parent pattern with the subgraph {(*x*, *y*), (*y*, *a*), (*a*, *b*)} which belongs to M7 (see Fig. 7)

Let us consider a directed graph *G* and pattern P1 of size *k* edges in *G*. Also, consider pattern P2 with *k* + 1 edges such that P2 contains P1 and an additional edge (*x*, *y*). It is required to show that P2 can be obtained from P1 by joining it with one of the seven basic building patterns. Since both P1 and P2 have connected graphs, let us assume that either *x* has an edge (*x*, *a*) or (*a*, *x*) or *y* has an edge (*y*, *a*) or (*a*, *y*) present in pattern P1. Fig. 16 illustrates these scenarios. First, the basic patterns M1, M2, and M3 (Fig. 7) are considered in the join operation. In these cases, either a copy of M1, {(*x*, *a*), (*x*, *y*)} or a copy of M3, {(*a*, *x*), (*x*, *y*)} or a copy of M3, {(*x*, *y*), (*y*, *a*)} or a copy of M2, {(*x*, *y*), (*a*, *y*)} will join with pattern P1 having a common edge (*x*, *a*) or (*a*, *x*) or (*y*, *a*) or (*a*, *y*), respectively, to produce pattern P2. These cases produce pattern P2 with *k* + 1 edges. This join, however, occurs only if the above subgraphs are included in the *F*2 counts of M1, M2, and M3. If all the above conditions fail, then there may exist four other possible scenarios, as shown in Fig. 16. If (*x*, *a*) and (*x*, *b*) exist then join a copy of the basic pattern M5 (Fig. 7), {(*x*, *y*), (*x*, *a*), (*x*, *b*)} with P1 to obtain P2. If (*b*, *a*) and (*a*, *x*) exist then join a copy of the basic pattern M7 (Fig. 7), {(*b*, *a*), (*a*, *x*), (*x*, *y*)} with P1 to obtain P2. If (*a*, *y*) and (*b*, *y*) exist then join a copy of the basic pattern M6 (Fig. 7), {(*a*, *y*), (*b*, *y*), (*x*, *y*)} with P1 to obtain P2. If (*y*, *a*) and (*a*, *b*) exist then join a copy of the basic pattern M7 (Fig. 7), {(*x*, *y*), (*y*, *a*), (*a*, *b*)} with P1 to obtain P2.

In summary, any pattern P2 with *k* + 1 edges can be constructed by joining pattern P1 with *k* edges (or *k* − 1 edges) with one of the basic building patterns.

Hence it can be concluded that the above four patterns in the undirected graph and seven patterns in the directed graph act as basic building patterns and any pattern present in the target network can be generated using the pattern‐join operation. The proposed algorithm uses *F*2 measure to compute the pattern frequency. Edge‐disjoint embeddings of a pattern are obtained by the MIS finding algorithm. A pattern is removed from the current set in two cases. (i) Pattern size matches with required motif size. (ii) Pattern frequency failed to cross the predefined frequency threshold. In the first case, the pattern is added to the output motif list and the second case is applicable as the *F*2 frequency measure satisfies the downward closure property. The algorithm terminates when no more patterns are present in the current set. The following section contains the pseudocode of the proposed motif discovery algorithm.

### 3.1 Pseudo‐code of motif discovery using the pattern‐join method

**Fig. 17:**
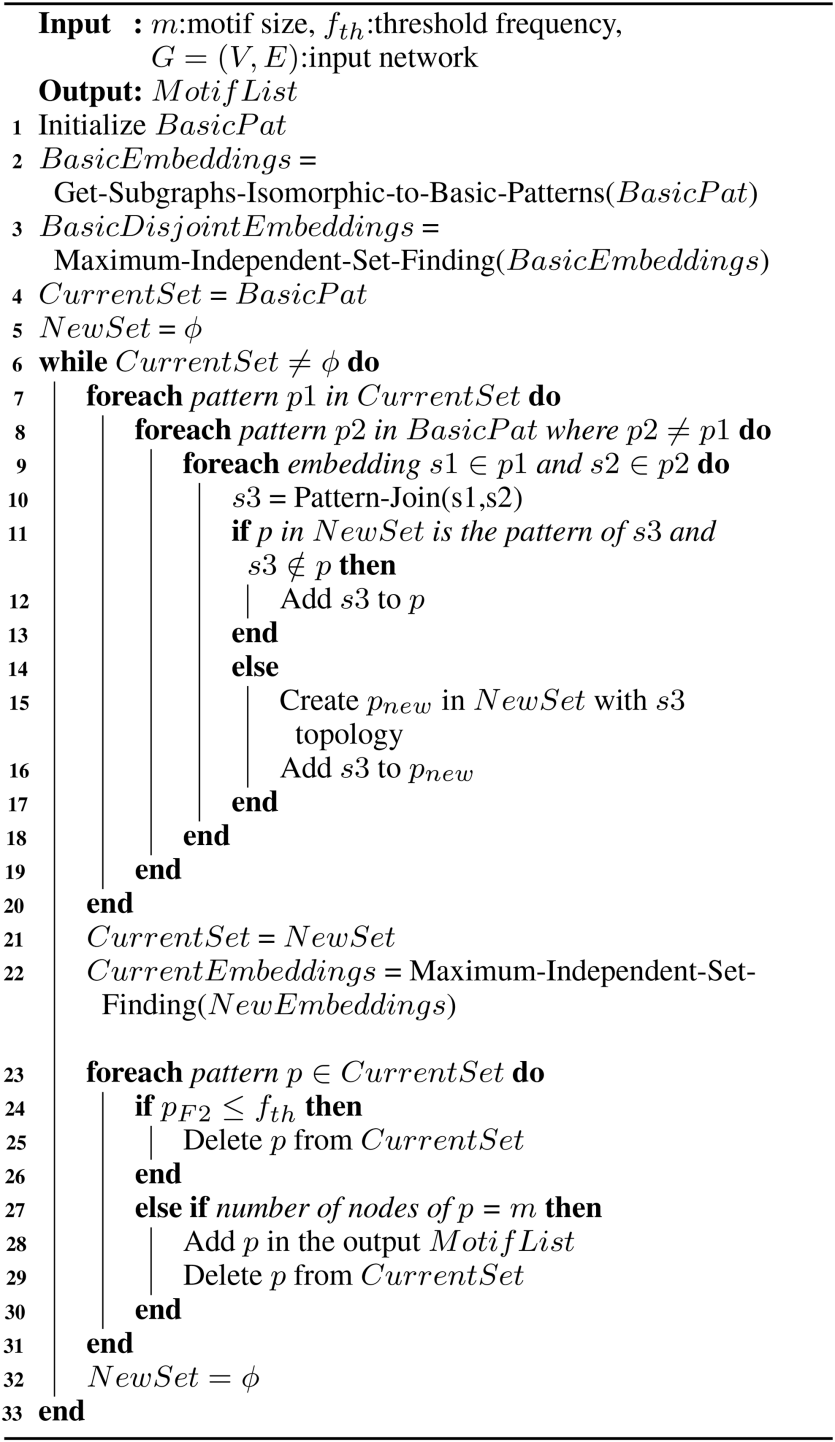
Algorithm 1: motif discovery using pattern‐join method

The pseudo‐code of the proposed method is represented by Algorithm 1 (see Fig. 17). The inputs to the algorithm are a graph *G*, motif size *m*, and the threshold frequency $f_{\text{th}}$. This algorithm first finds all the embeddings of basic building patterns (Line 2). The detail of this process is present in Sections 3.2 and 3.3. Then the algorithm extracts the edge‐disjoint embeddings of each pattern (Line 3) using an MIS finding algorithm, which is explained in Section 3.4. The current set of patterns is initialised to four basic patterns in the case of the undirected graph and seven basic patterns in the case of the directed graph. The new set is initialised to an empty set. The size of the current motif set increases in each successive iteration.

This algorithm joins the instances of each sub‐graph present in the current set with the instances of basic building pattern set (Line 10). Two subgraphs can be joined if they share at least one edge and joining of subgraphs belonging to the same pattern is not allowed. Either a new pattern is created or an existing pattern is generated as a result of joining two subgraphs (Lines 11–17). The detail of the pattern‐join operation is explained in Section 3.5. In Line‐22, the overlapping instances of each pattern in the new set are removed from the MIS finding algorithm and the result is saved in the current set for the next iteration. The patterns which failed to satisfy the frequency threshold are removed from the current set (Lines 24–26). The algorithm stores the patterns of target motif size in the output motif list and then delete it from the current set (Lines 27–30). When the current set becomes empty, the algorithm terminates (Line 6).

### 3.2 Embeddings of basic patterns for undirected graph

Following procedures are adopted to find the embeddings of basic patterns M1–M4, as shown in Fig. 6:

- To find out embeddings of pattern M1, select all possible combinations of any two edges connected to each node $v_{i}\in V$ of a network *G*. The number of such embeddings will be $\sum_{v_{i}\in V}\left(\begin{array}{c} d(v_{i})\\ 2 \end{array}\right)$, where d(v) represents the degree of a vertex *v*.
- To find out embeddings of pattern M2, for each edge $(v_{i},v_{j})\in E$ of the network *G*, select all possible vertices $v_{k}\in V$ which are connected to both $v_{i}$ and $v_{j}$ for all k≠i,j. The upper bound of the number of such embeddings will be $\sum_{(v_{i},v_{j})\in E}\min(d(v_{i}),d(v_{j}))-1$.
- To find out embeddings of pattern M3, select all possible combinations of any three edges connected to each node $v_{i}\in V$ of a network *G*. The number of such embeddings will be $\sum_{v_{i}\in V}\left(\begin{array}{c} d(v_{i})\\ 3 \end{array}\right)$.
- To find out embeddings of pattern M4, for each edge $(v_{i},v_{j})\in E$ of the network *G*, select any two vertices $v_{3},v_{4}\in V$ where $v_{3}$ is adjacent to $v_{i}$ and $v_{4}$ is adjacent to $v_{j}$, but they are not adjacent to each other. The number of such embeddings will be less than $\sum_{(v_{i},v_{j})\in E}d(v_{i})d(v_{j})$.

### 3.3 Embeddings of basic patterns for a directed graph

Following procedures are adopted to find the embeddings of basic patterns M1–M7, as shown in Fig. 7:

- To find out embeddings of pattern M1, select all possible combinations of any two outgoing edges from each node $v_{i}\in V$ of a network *G*. The number of such embeddings will be $\sum_{v_{i}\in V}\left(\begin{array}{c} d_{o}(v_{i})\\ 2 \end{array}\right)$, where $d_{o}(v)$ represents the out‐degree of the vertex *v*.
- To find out embeddings of pattern M2, select all possible combinations of any two incoming edges to each node $v_{i}\in V$ of a network *G*. The number of such embeddings will be $\sum_{v_{i}\in V}\left(\begin{array}{c} d_{i}(v_{i})\\ 2 \end{array}\right)$, where $d_{i}(v)$ represents the in‐degree of the vertex *v*.
- To find out embeddings of pattern M3, select all possible combinations of an incoming edge and an outgoing edge for each node $v_{i}\in V$ of a network *G*. The number of such embeddings will be $\sum_{v_{i}\in V}d_{o}(v_{i})d_{i}(v_{i})$.
- To find out embeddings of pattern M4, select all possible combinations of an outgoing edge ($v_{i}\to v_{2}$) and an incoming edge ($v_{i}\leftarrow v_{3}$) for each node $v_{i}\in V$ of the network *G* then check for an edge ($v_{2}\to v_{3}$). The number of such embeddings will be less than $\sum_{v_{i}\in V}d_{o}(v_{i})d_{i}(v_{i})$.
- To find out embeddings of pattern M5, select all possible combination of any three outgoing edges from each node $v_{i}\in V$ of a network *G*. The number of such embeddings will be $\sum_{v_{i}\in V}\left(\begin{array}{c} d_{o}(v_{i})\\ 3 \end{array}\right)$.
- To find out embeddings of pattern M6, select all possible combinations of any three incoming edges to each node $v_{i}\in V$ of a network *G*. The number of such embeddings will be $\sum_{v_{i}\in V}\left(\begin{array}{c} d_{i}(v_{i})\\ 3 \end{array}\right)$.
- To find out embeddings of pattern M7, select an edge ($v_{i}\to v_{j}$) then select all possible combinations of an incoming edge to $v_{i}$ ($v_{i}\leftarrow v_{3}$) and an outgoing edge from $v_{j}$ ($v_{j}\to v_{4}$) of the network *G* then check the condition ($v_{3}\neq v_{4}$). The number of such embeddings will be $\sum_{(v_{i},v_{j})\in E}d_{i}(v_{i})d_{o}(v_{j})$.

### 3.4 MIS finding algorithm

**Fig. 18:**
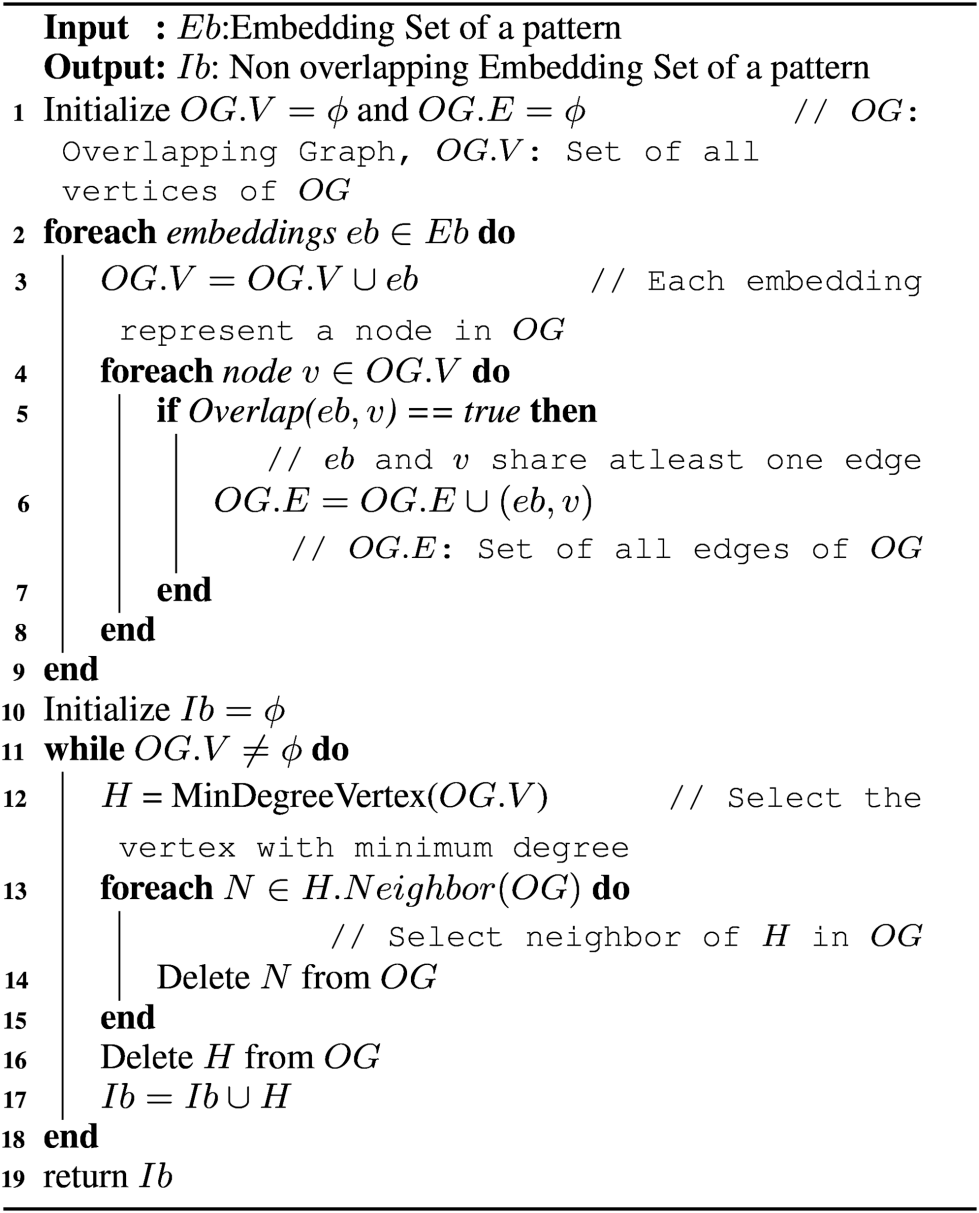
Algorithm 2: MIS finding algorithm

This algorithm has two phases, (i) construction of overlap graph, (ii) finding an MIS of non‐overlapping subgraphs. Algorithm 2 (see Fig. 18) constructs the overlap graph in Lines 1–9. Each node in the overlap graph represents an embedding of a pattern in the target network. Overlapped embeddings of a pattern in the target network are connected through edges in the overlap graph. Lines 4–8 perform this task. Once the overlap graph is created, a node with the minimum number of neighbours is selected from the overlap graph (Line 12). The embedding corresponding to this node is added in the edge‐disjoint set (Line 17). Then, this node is deleted with its neighbour from the overlap graph (Lines 13–16). Then this algorithm updates the degree of all the nodes which were connected to deleted nodes. The process of picking and shrinking continue until the overlap graph becomes empty.

### 3.5 Pattern‐join operation

**Fig. 19:**
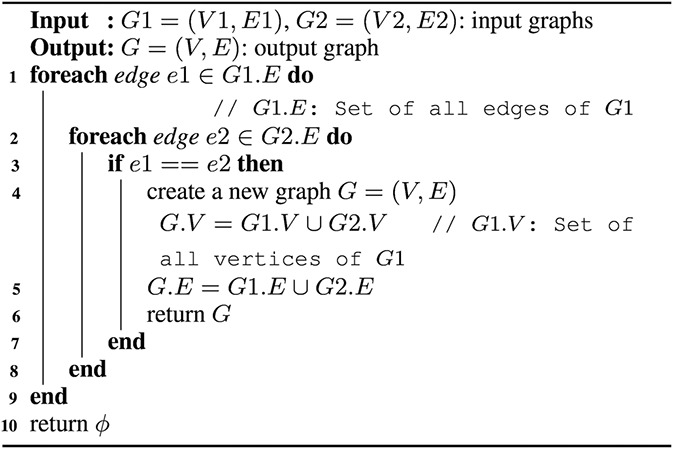
Algorithm 3: pattern‐join operation

In pattern‐join operation, two subgraphs of a given network join only if they share at least one edge. Algorithm 3 (see Fig. 19) contains the pseudo‐code of the joining procedure. This algorithm checks the existence of the same edge in both the subgraphs from Lines 1 to 3. A new graph *G*, which is supergraph of both G1 and G2, is created when an edge appeared in both the subgraphs. This task is performed in Lines 4–6. When there is no common edge found in the subgraphs, Line‐10 returns an empty graph.

### 3.6 Computational complexity

In this section, the computational complexity of each module is formally analysed.

*Finding embeddings of basic building patterns:* For an undirected graph, the computational complexity of this step can be expressed as

$$\begin{array}{rl} T(n) & =\sum_{v_{i}\in V}\left(\begin{array}{c} d(v_{i})\\ 2 \end{array}\right)+\sum_{(v_{i},v_{j})\in E}d(v_{i})d(v_{j})\\ & \;+\sum_{v_{i}\in V}\left(\begin{array}{c} d(v_{i})\\ 3 \end{array}\right)+\sum_{(v_{i},v_{j})\in E}d(v_{i})d(v_{j})\\ & =O\left(\sum_{v_{i}\in V}d{\left(v_{i}\right)}^{2}+\sum_{(v_{i},v_{j})\in E}d(v_{i})d(v_{j})+\sum_{v_{i}\in V}d{\left(v_{i}\right)}^{3})\right) \end{array}$$

For a directed graph, the computational complexity of this step can be expressed as

$$\begin{array}{rl} T(n) & =\sum_{v_{i}\in V}\left(\begin{array}{c} d_{o}(v_{i})\\ 2 \end{array}\right)+\sum_{v_{i}\in V}\left(\begin{array}{c} d_{i}(v_{i})\\ 2 \end{array}\right)+\sum_{v_{i}\in V}d_{o}(v_{i})d_{i}(v_{i})\\ & \;+\sum_{v_{i}\in V}d_{o}(v_{i})d_{i}(v_{i})+\sum_{v_{i}\in V}\left(\begin{array}{c} d_{o}(v_{i})\\ 3 \end{array}\right)\\ & \;+\sum_{v_{i}\in V}\left(\begin{array}{c} d_{i}(v_{i})\\ 3 \end{array}\right)+\sum_{(v_{i},v_{j})\in E}d_{i}(v_{i})d_{o}(v_{j})\\ & =O\left(\sum_{v_{i}\in V}d{\left(v_{i}\right)}^{2}+\sum_{(v_{i},v_{j})\in E}d(v_{i})d(v_{j})+\sum_{v_{i}\in V}d{\left(v_{i}\right)}^{3}\right) \end{array}$$

The worst‐case scenario happens when $d(v_{i})=O(n)$. In this scenario, the computational complexity of this step becomes $O(n^{4})$.

*MIS finding algorithm:* Let *m* represents the number of overlapping embeddings. For basic building patterns $m=O(n^{4})$. However, the value of *m* reduces significantly in the successive iteration. The computational complexity of constructing the overlapping graph is $O(m^{2})$. A min‐heap is created based on their degree from the nodes of the overlapping graph. The cost of constructing the min‐heap is O(m). Disjoint embeddings are obtained by deleting the nodes one by one from the min‐heap and adjusting the rest of the nodes. This process has complexity equal to O(mlog(m)).

*Pattern‐join operation:* In this step, we analyse the complexity of join iteration. Let $x_{i}$ denotes the number of patterns in iteration *i*. For an undirected graph, $x_{i}$ starts at 4 and this starts at 7 in the case of a directed graph. In each iteration, the size of the pattern is increased by 1 or 2 edges. The initial size is either 2 or 3. Thus, the minimum size of each pattern at the *i*th iteration is *i* + 2 and the number of non‐overlapping embeddings of a pattern is at most $\frac{\left|E\right|}{i+2}$, where |E| represents the number of edges present in the input network. The total number of disjoint embeddings of all the basic building patterns for both undirected and directed graph is O(|E|). During joining, embeddings of each pattern joined with all the embeddings of basic building patterns. Thus, the total number of join operations performed at iteration *i* is $O\left(|E|\frac{\left|E\right|}{i+2}x_{i}\right)$. For each join, resulting subgraph is compared against each pattern in that iteration and the cost of this operation is $O(x_{i})$. The complexity of removing duplicate embeddings is $O\left(\log\frac{\left|E\right|}{i+2}\right)$. Collectively, the complexity of performing all the joins at iteration *i* is obtained by multiplying the above three complexities. This is computed as $O\left(|E|\frac{\left|E\right|}{i+2}x_{i}x_{i}\log\frac{\left|E\right|}{i+2}\right)$ which equals $O\left(x_{i}^{2}\frac{|E{|}^{2}}{i+2}\log\frac{\left|E\right|}{i+2}\right)$.

## 4 Results and discussion

The performance of the proposed motif discovery algorithm is evaluated on a real dataset for both undirected and directed networks. The runtime and the number of significant motifs are two primary criteria for evaluation of the proposed motif discovery algorithm. The runtime of the proposed motif discovery algorithm is compared against existing algorithms by varying both motif size and network size. Frequency measure *F*2 is used to compute motif frequency and *z*‐score is used to measure the statistical significance of the identified network motif. The performance of the proposed algorithm is compared against MFinder, ESU, Grochow–Kellis, and MODA algorithms.

### 4.1 Data set and computational environment

The proposed algorithm is tested in both undirected and directed networks. The transcription regulatory network of *Escherichia coli* (Eco) [28] is used for the directed network. This database contains 578 interactions between 116 TFs and 423 operons. The data is presented in a simple interaction format (SIF) with three columns. For the undirected network, MIPS mammalian protein–protein interaction database of *Saccharomyces cerevisiae* (Sce) [29] and Molecular INTeraction (MINT) database of Human herpesvirus‐8 (Hhv8) [30] is used. The Sce network contains 1815 interactions among 858 proteins, and Hhv8 network contains only 170 interactions among 92 proteins. The proposed algorithm is implemented in C++ with Intel(R) Xeon(R) E5‐2670 Processor 2.3 GHz CPU, 64 GBs of main memory running Redhat Linux operating system.

### 4.2 Performance evaluation

The performance of the proposed motif discovery algorithm is evaluated based on runtime, statistically significant motifs and *z*‐score of most abundant motifs.

#### 4.2.1 Runtime

**Fig. 20:**
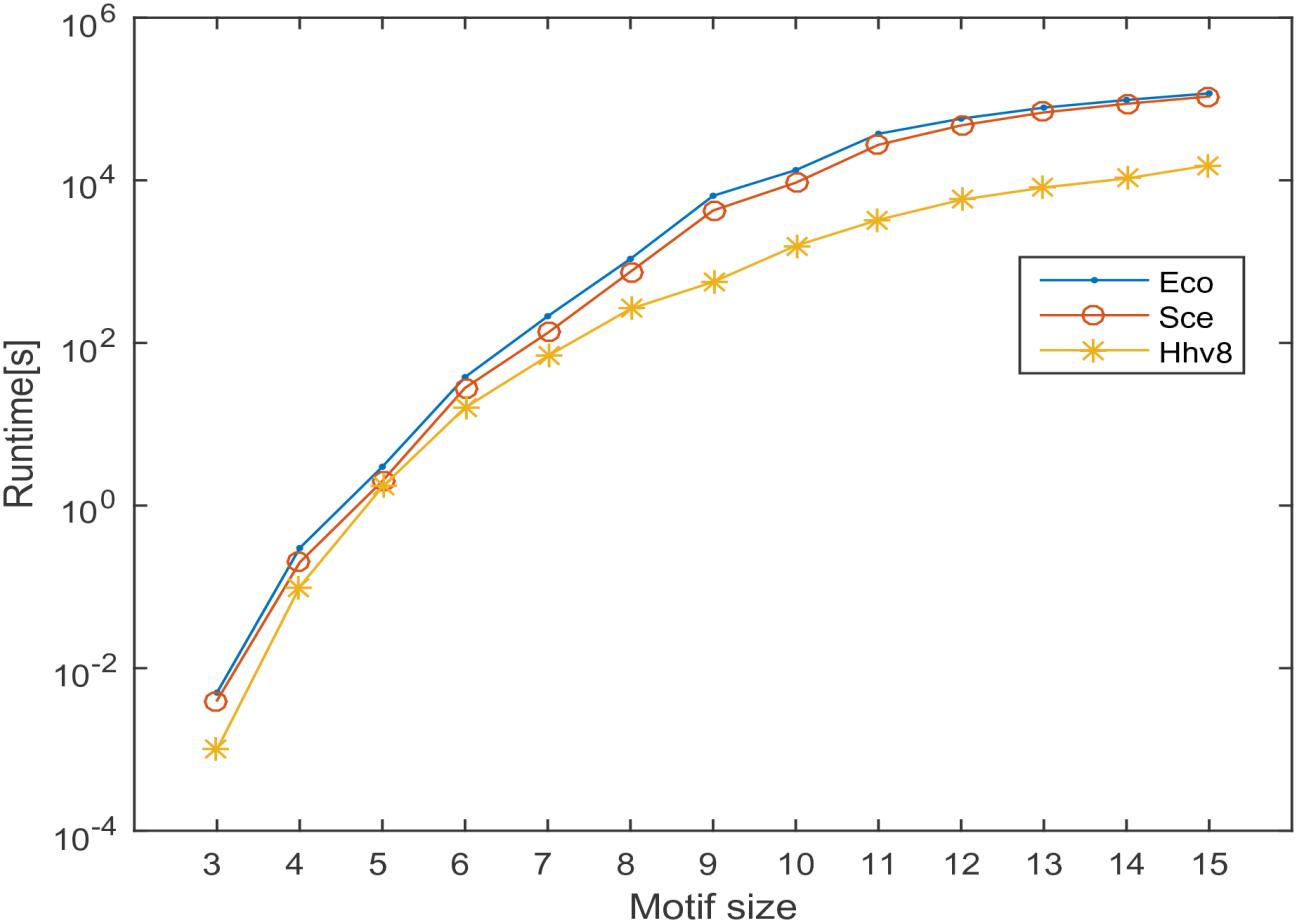
Runtime of the pattern‐join method by varying motif size on a real network of Escherichia coli (Eco), Saccharomyces cerevisiae (Sce) and Human herpesvirus 8 (Hhv8). The x‐axis indicates the motif size and the y‐axis shows the runtime in seconds

In this section, the runtime of the proposed motif discovery algorithm is computed on directed and undirected biological networks specified above. During this computation, the frequency threshold is set as 5% of the size of the network and the threshold for *z*‐score is set as 2. The *F*2 measure is used to compute motif frequency. The effect of motif size on the runtime is observed by varying the motif sizes from 3 to 15 and the results obtained are shown in Fig. 20. The behaviour of the result is a clear indication of the scalability of the proposed algorithm concerning the motif size. The proposed algorithm takes only a few minutes to run for motif sizes 3–10 for both directed and undirected networks and it is limited to a few hours for motif sizes 11–15. For higher motif size, the runtime is influenced by the motif size. This behaviour is observed due to the number of alternative patterns increases exponentially toconcerning motif size. Irrespective of this limitation the proposed method can discover motifs up to size‐15 within a practical runtime.

#### 4.2.2 Statistically and biologically significant motifs

**Fig. 21:**
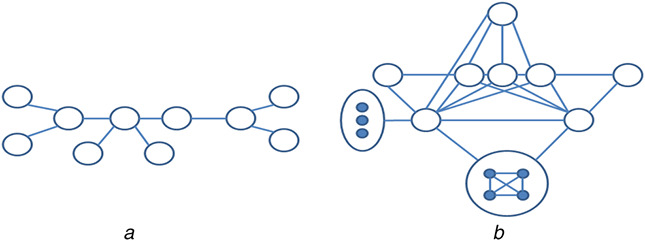
*Motif of 10 nodes in the left found in the PPI network of Human herpesvirus‐8 [* *22* *] and a motif of 15 nodes in the right found in the PPI network of S. cerevisiae [* *31* *]*

Table 1 contains the number of significant motifs found by setting the frequency threshold as 5% of the size of the network. The experiment is performed on the transcription regulatory network of *Escherichia coli* (Eco) and protein–protein interaction network of *Saccharomyces cerevisiae* (Sce), and Human herpesvirus‐8 (Hhv8). The identified motifs are statistically significant as they are over‐represented in the target network. Some of these motifs may not be biologically significant. One of the biologically significant motifs found in the PPI network of Human herpesvirus‐8 is shown in Fig. 21. This network motif of 10 nodes causes Kaposi sarcoma disease. Another biologically significant motif found in *S. cerevisiae* consists of 15 nodes, as shown in Fig. 21. This network motif is responsible for transcriptional machinery and cell‐cycle regulation in the said network.

**Table 1:** Number of significant motifs in transcription regulatory network of *Escherichia coli* (Eco) and protein–protein interaction network of *Saccharomyces cerevisiae* (Sce) and Human herpesvirus‐8 (Hhv8)

Motif size	3	4	5	6	7	8	9	11	13	15
Eco	2	7	12	46	107	759	2932	6025	7516	8327
Sce	0	4	9	38	92	588	2209	5218	6581	7916
Hhv8	1	6	10	52	104	685	2861	5914	7096	8152

#### 4.2.3 *z*‐score that represent significant of the most abundant motif

In this section, the statistical significance of the most abundant motif is discussed across the three biological networks. The statistical significance of the most abundant motif of a given size is computed concerning the abundance of the same pattern in a set of random graphs. The mathematical parameter used for this purpose is termed as *z*‐score. A higher value of *z*‐score represents a more significant motif. Typically the threshold value is taken as 2. Table 2 presents the *z*‐score of the most abundant motif across three biological networks for seven motif sizes (*m* = 3, 5, 7, 9, 11, 13, 15). In Table 2, it is observed that the *z*‐score of small motifs (i.e. up to *m* = 7) is not so high as compared to large motifs. However, as motif size increases (i.e. *m* = 9–15), the frequency gap between the most abundant motif in the real network and the random networks becomes highly significant. This implies the statistical significance of large motifs as compared to small motifs.

**Table 2:** z‐scores that represent the significance of the most abundant motif against 100 random networks in each specified network using seven motif sizes

Motif size	3	5	7	9	11	13	15
Eco	2.32	3.56	5.29	6.63	6.24	5.10	7.21
Sce	2.73	4.31	4.45	8.22	6.54	7.58	9.29
Hhv8	4.91	6.12	5.24	7.25	9.47	8.58	10.27

### 4.3 Runtime comparison with existing methods by varying motif size

**Fig. 22:**
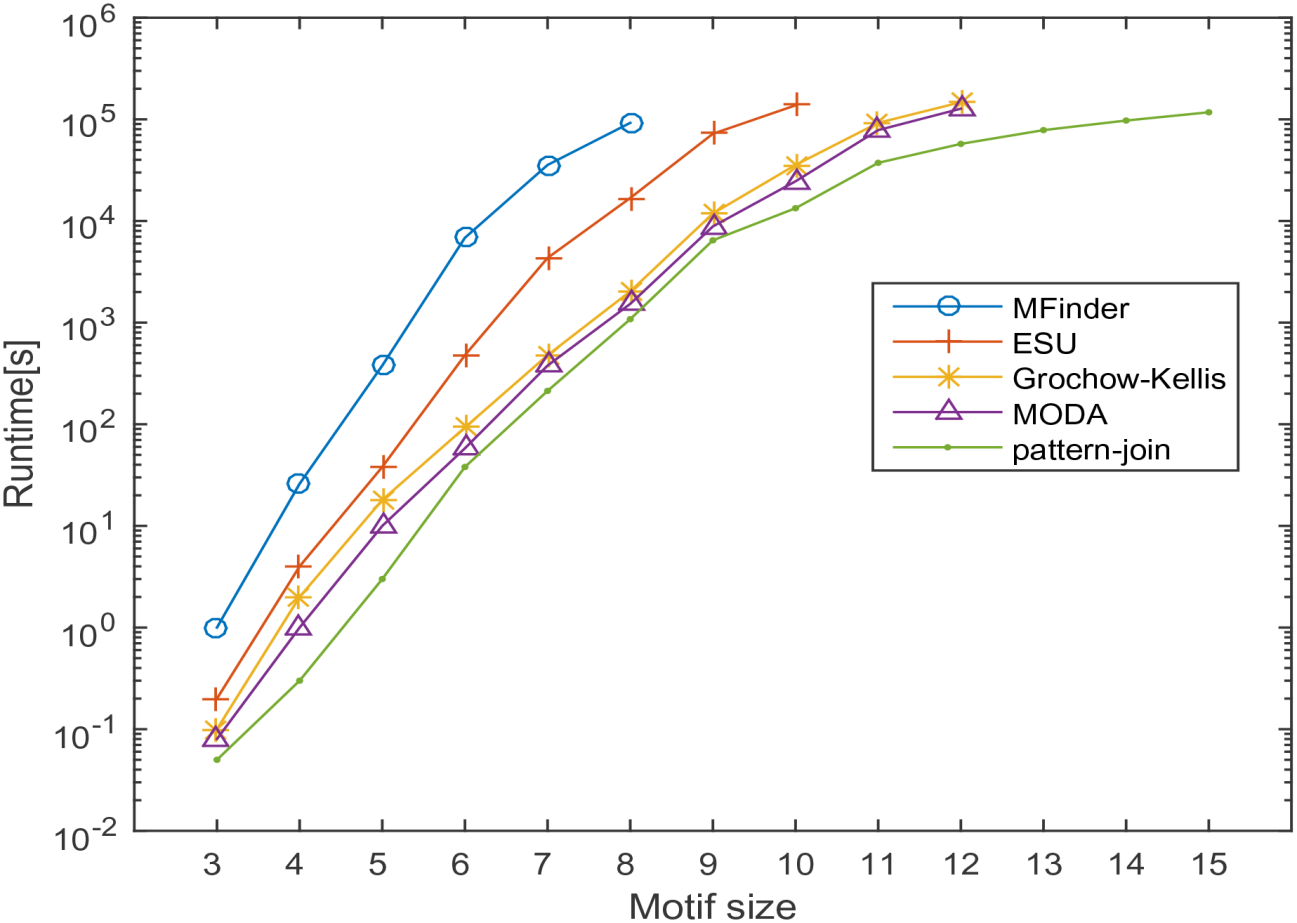
Runtime of MFinder, ESU, Grochow–Kellis, MODA and pattern‐join method on a real network of Escherichia coli (Eco). The x‐axis indicates the motif size and the y‐axis shows the runtime in seconds

**Fig. 23:**
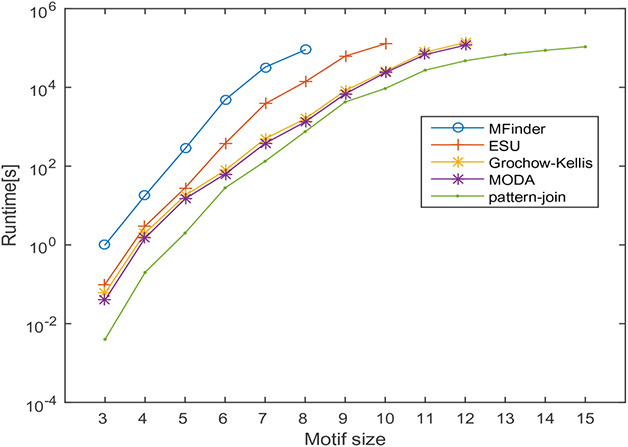
Runtime of MFinder, ESU, Grochow–Kellis, MODA and pattern‐join method on a real network of Saccharomyces cerevisiae (Sce). The x‐axis indicates the motif size and the y‐axis shows the runtime in seconds

**Fig. 24:**
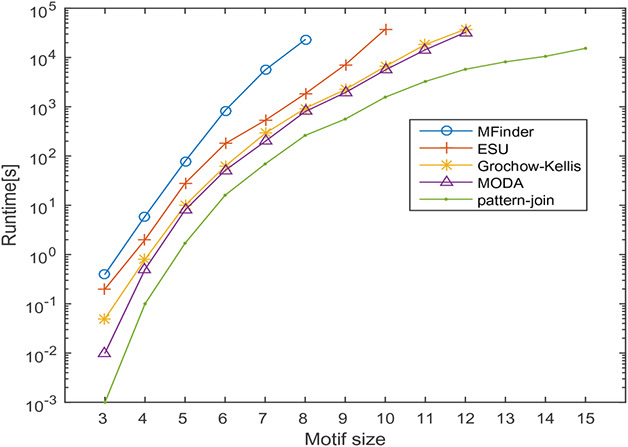
Runtime of MFinder, ESU, Grochow–Kellis, MODA and pattern‐join method on a real network of Human herpesvirus‐8 (Hhv8). The x‐axis indicates the motif size and the y‐axis shows the runtime in seconds

In this section, the runtime of the proposed motif discovery algorithm is measured on the transcription regulatory network of *Escherichia coli* and protein–protein interaction network of *Saccharomyces cerevisiae* and Human herpesvirus‐8. The runtime of the proposed method is compared against MFinder, ESU, Grochow–Kellis, and MODA algorithms. The effect of varying motif size on the runtime of the algorithms is observed by varying motif sizes from 3 to 15. In this experiment, the frequency threshold is set as 5% of the size of the network. The effect of motif size on the runtime is observed the results obtained are shown in Figs. 22, 23, 24.

Significant factors affecting the runtime are the number of alternative motif topologies and subgraph isomorphism check. Despite these factors, the runtime of the proposed algorithm increases in polynomial order concerning motif size. MFinder and ESU can find out motifs up to size‐8 and size‐10, respectively, in a practical time bound. Grochow–Kellis and MODA can find out motifs up to size‐12 in a practical time bound. The proposed algorithm can find out motifs up to size‐15. The behaviour of the result is a clear indication of the scalability of the proposed algorithm concerning motif size. The proposed algorithm takes only a few minutes to run for motif sizes 3–10, and it is limited to a few hours for motif sizes 11–15.

### 4.4 Runtime comparison with existing methods by varying network size

**Fig. 25:**
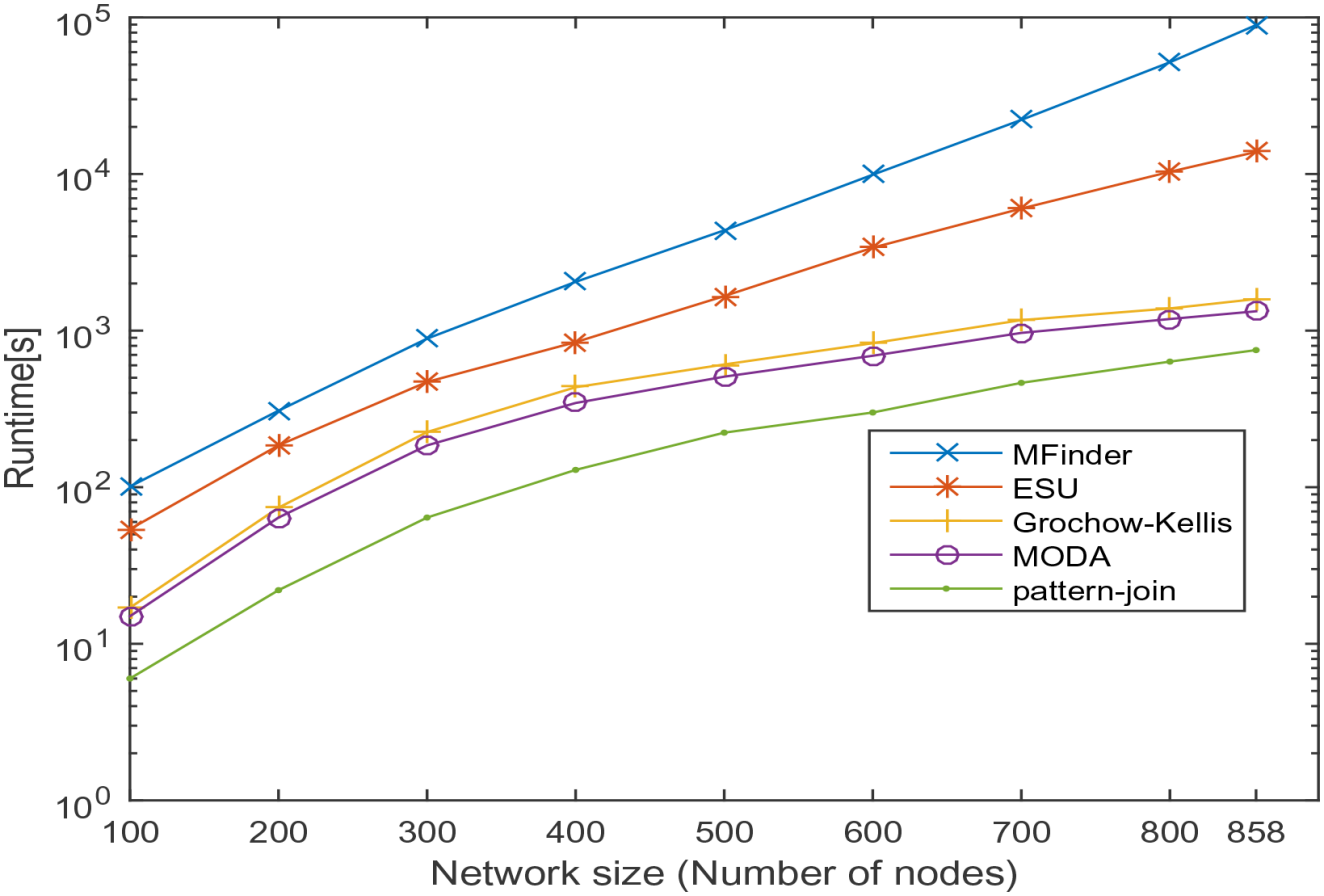
Runtime of MFinder, ESU, Grochow–Kellis, MODA, and pattern‐join for PPI sub‐network of Saccharomyces cerevisiae (Sce). Network size is varied along the x‐axis from 100 to 858. Runtime is measured in seconds. Motif size is taken as 8

**Fig. 26:**
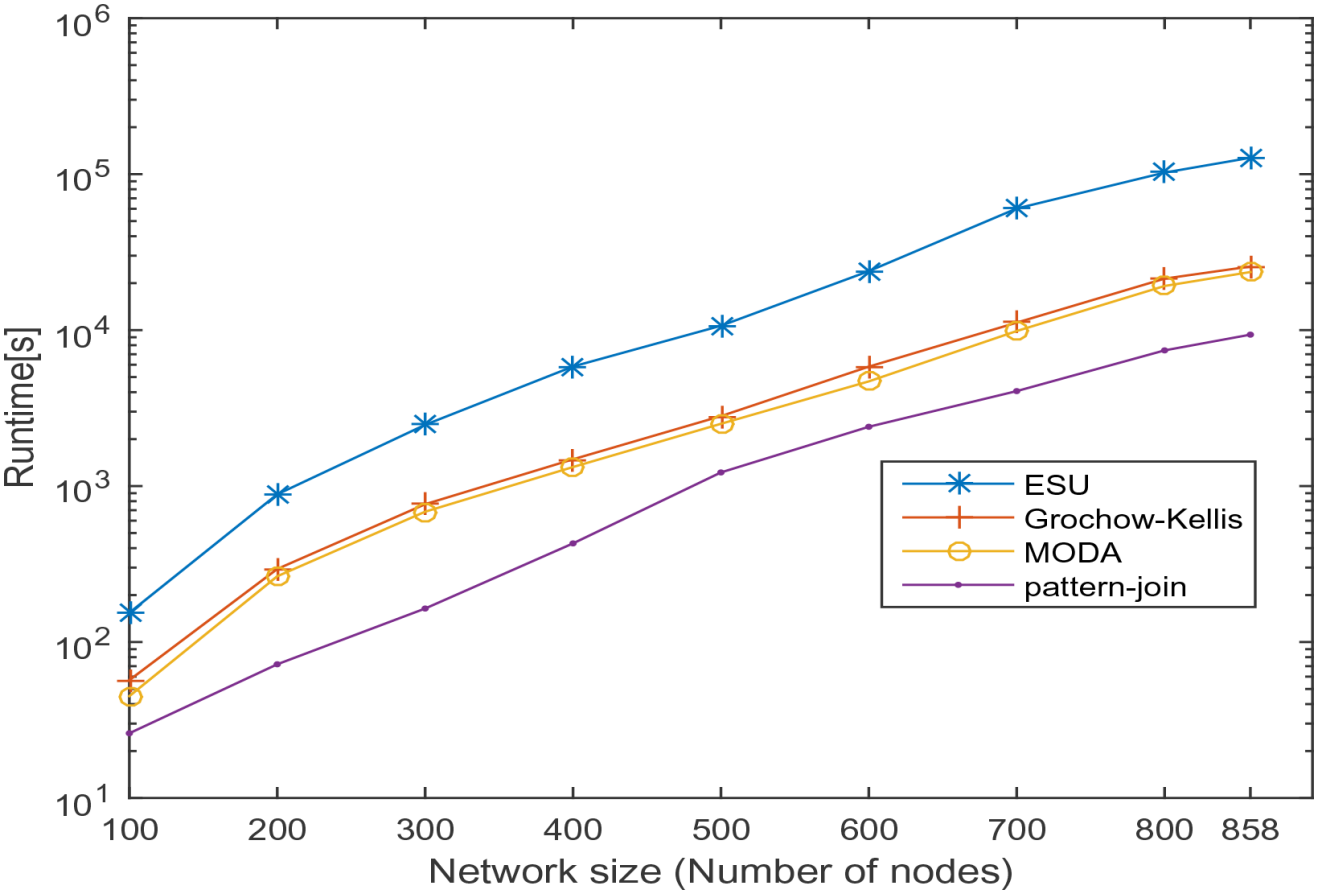
Runtime of ESU, Grochow–Kellis, MODA, and pattern‐join for PPI sub‐network of Saccharomyces cerevisiae (Sce). Network size is varied along the x‐axis from 100 to 858. Runtime is measured in seconds. Motif size is taken as 10

**Fig. 27:**
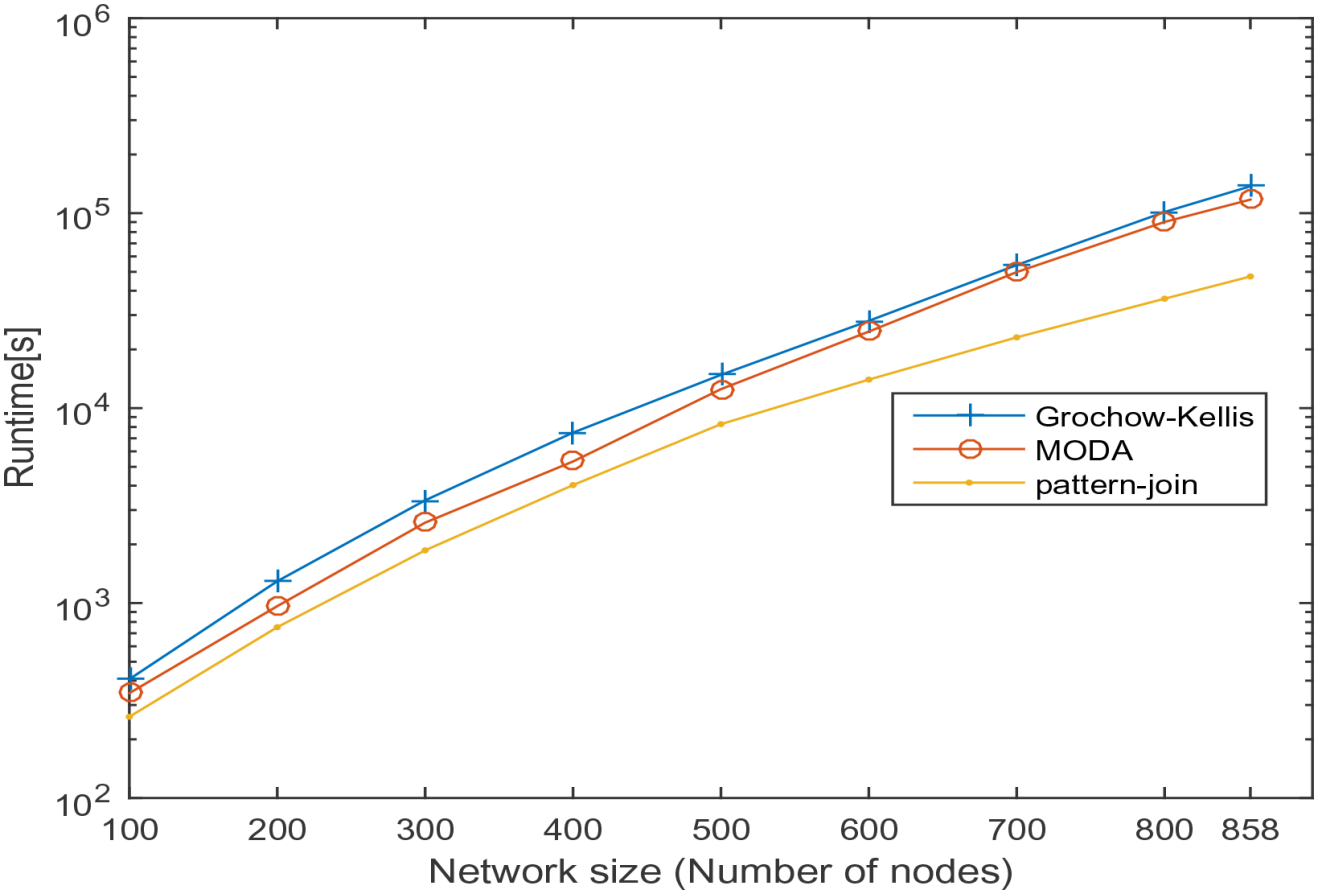
Runtime of Grochow–Kellis, MODA, and pattern‐join for PPI sub‐network of Saccharomyces cerevisiae (Sce). Network size is varied along the x‐axis from 100 to 858. Runtime is measured in seconds. Motif size is taken as 12

**Fig. 28:**
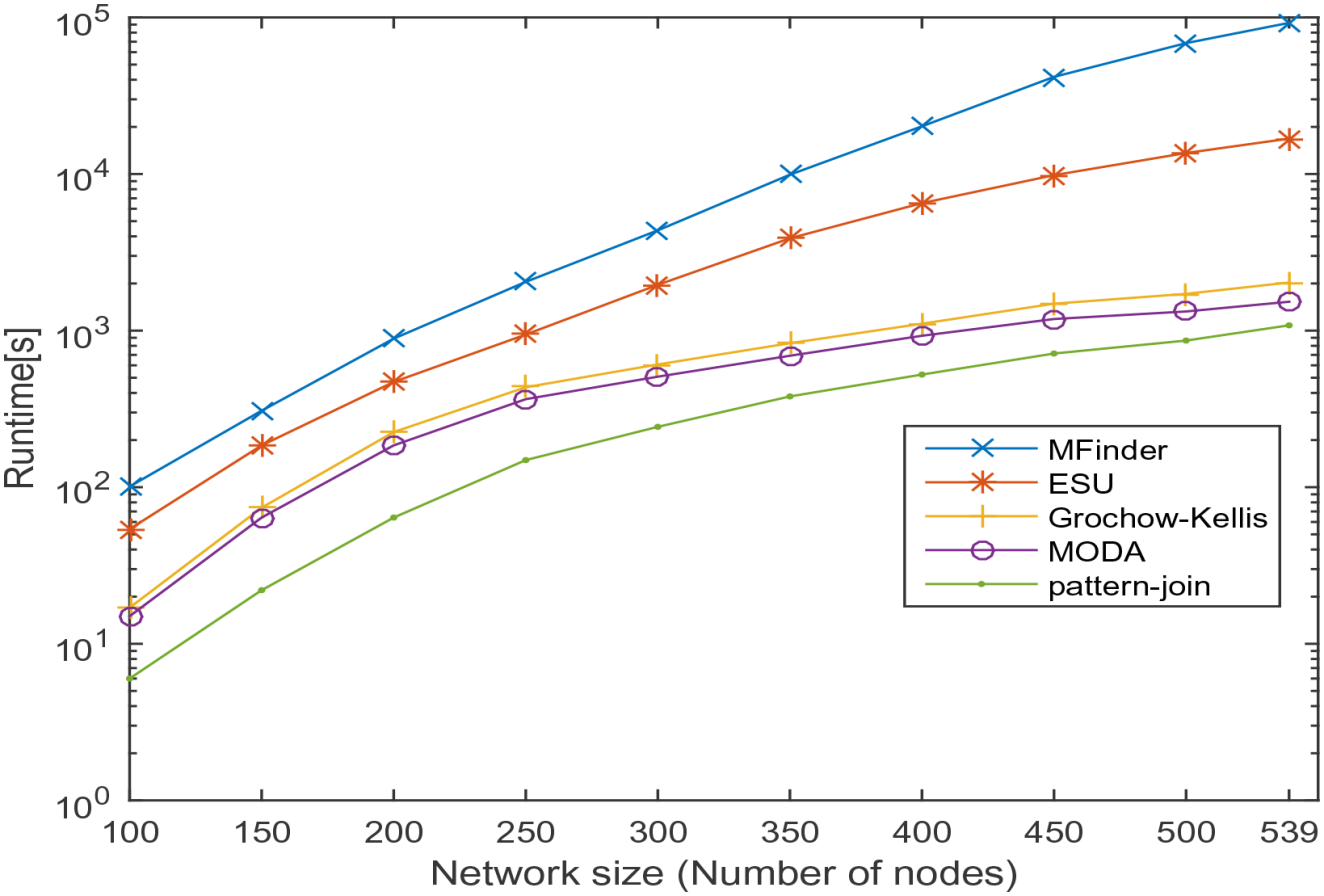
Runtime of MFinder, ESU, Grochow–Kellis, MODA, and pattern‐join for PPI sub‐network of Escherichia coli (Eco). Network size is varied along the x‐axis from 100 to 539. Runtime is measured in seconds. Motif size is taken as 8

**Fig. 29:**
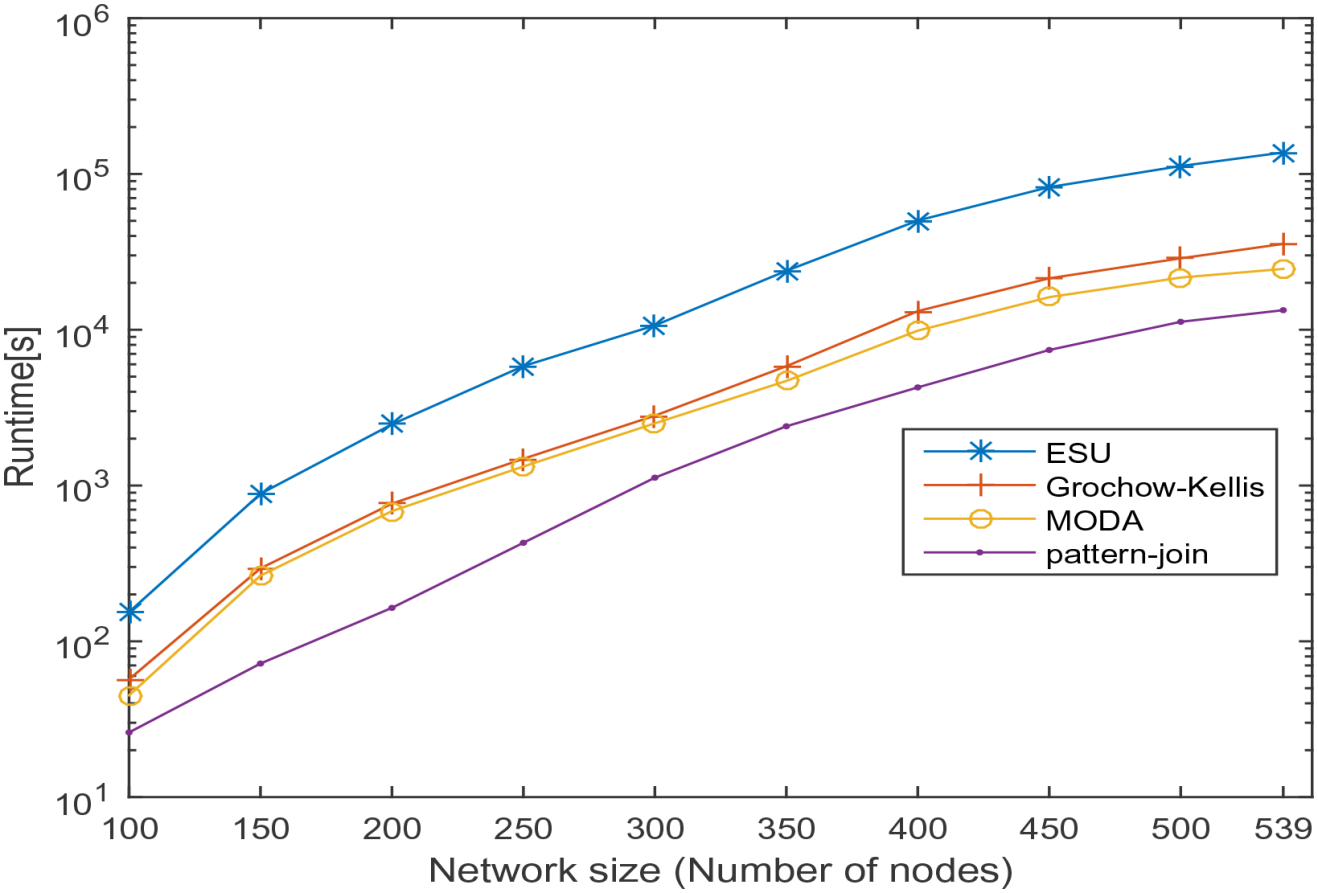
Runtime of ESU, Grochow–Kellis, MODA, and pattern‐join for PPI sub‐network of Escherichia coli (Eco). Network size is varied along the x‐axis from 100 to 539. Runtime is measured in seconds. Motif size is taken as 10

**Fig. 30:**
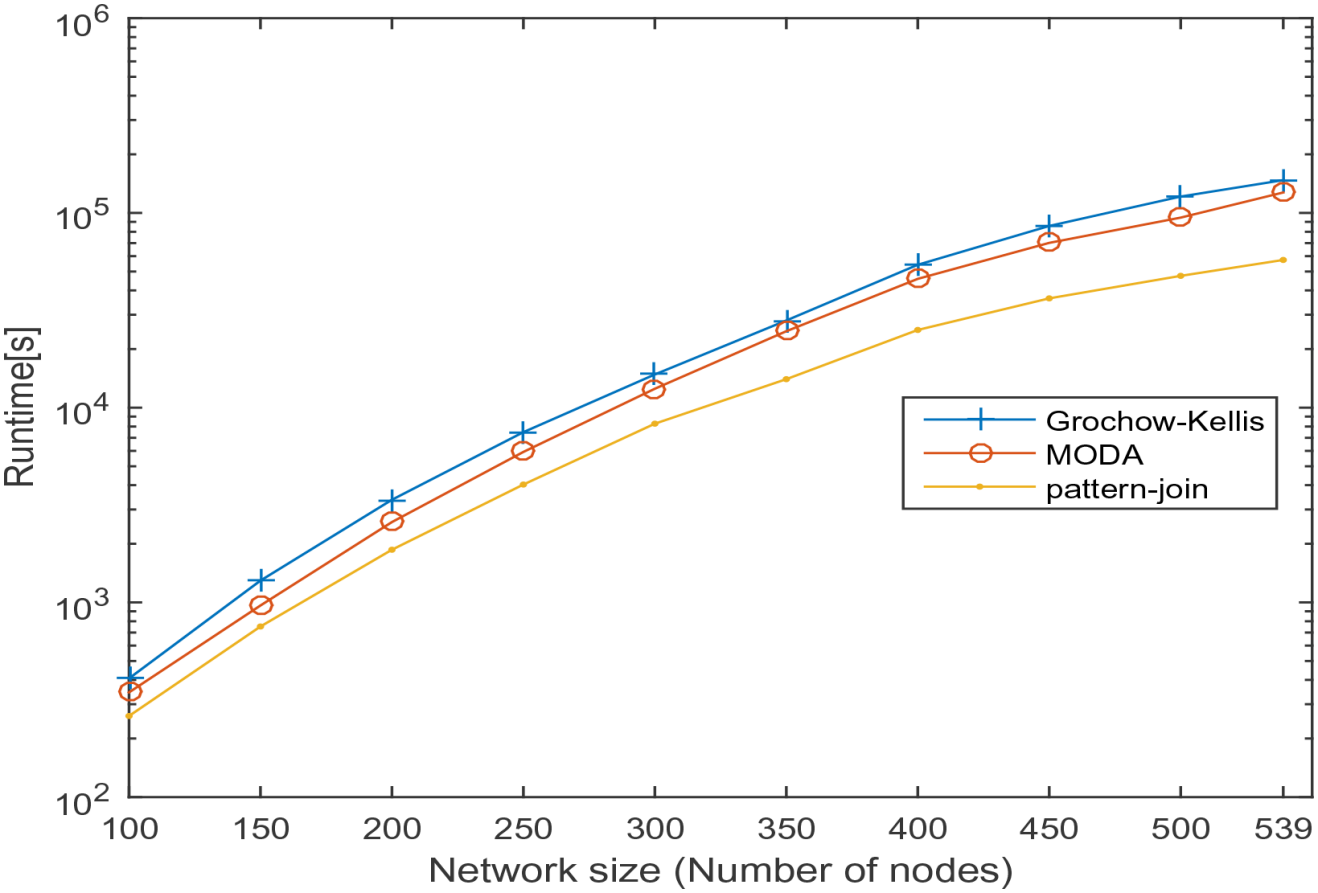
Runtime of Grochow–Kellis, MODA, and pattern‐join for PPI sub‐network of Escherichia coli (Eco). Network size is varied along the x‐axis from 100 to 539. Runtime is measured in seconds. Motif size is taken as 12

In this section, undirected networks of varying size from 100 to 858 and directed networks of varying size from 100 to 539 are generated from a real PPI network of *Saccharomyces cerevisiae* (Sce) and transcription regulatory network of *Escherichia coli* (Eco), respectively. The node set is selected in random order, and 10 sets are prepared for each size. The number of nodes and the average number of interactions is shown in Tables 3 and 4 for undirected and directed networks, respectively. The average runtime is reported for each subnetwork obtained by repeating the experiment ten times, once for each set. The simulation results indicate that the proposed method is reliable and computationally feasible for the large network. The runtime of the proposed method for motif sizes 8, 10 and 12 is observed by varying network size, as shown in Tables 3 and 4. The measured runtime is compared with MFinder, ESU, Grochow–Kellis, and MODA as applicable. The results obtained are shown in Figs. 25, 26, 27, 28, 29, 30. The results indicate that the proposed method is scalable as compared to existing methods.

**Table 3:** Each column represents a subset real PPI network of *Saccharomyces cerevisiae* (Sce)

number of nodes	100	200	300	400	500	600	700	800	858
number of interactions	97	219	384	580	807	981	1224	1653	1815

**Table 4:** Each column represents a subset of the transcription regulatory network of *Escherichia coli* (Eco)

number of nodes	100	200	300	400	500	539
number of interactions	87	192	294	407	523	578

## 5 Conclusion

In this paper, a motif discovery algorithm using pattern join method is proposed. The proposed method discovers the edge‐disjoint embeddings of frequent patterns in two steps. Initially, it finds the embeddings of a pattern by joining its parent pattern with the basic building pattern. Finally, the edge‐disjoint embeddings are obtained by applying the MIS finding algorithm. Isomorphic check through canonical representation significantly reduced the computational time of the proposed algorithm. Irrespective of the exponential growth of the number of patterns concerning size, this algorithm does not expand too much in the successive iteration as most of the patterns failed to cross the threshold frequency and not consider for the next iteration. Hence the runtime does not increase exponentially. The runtime of the proposed algorithm is evaluated by varying motif size and network size. Our implementation results indicate that the proposed algorithm is significantly faster than the existing motif discovery algorithms, and it can able to discover large motifs up to size‐15 within a few hours. In this proposed method, the *F*2 frequency measure is used to find edge‐disjoint subgraphs. A similar approach can be used to find completely disjoint subgraphs by using the *F*3 frequency measure, which is taken as a future work of this paper.
